# Downregulation of MEG3 promotes neuroblastoma development through FOXO1-mediated autophagy and mTOR-mediated epithelial-mesenchymal transition

**DOI:** 10.7150/ijbs.48126

**Published:** 2020-10-03

**Authors:** Mujie Ye, Hong Lu, Weitao Tang, Tianrui Jing, Shiyu Chen, Meng Wei, Jingjing Zhang, Jing Wang, Jing Ma, Duan Ma, Kuiran Dong

**Affiliations:** 1 Department of Pediatric Surgery, Children's Hospital of Fudan University, Shanghai, 201102, China.; 2 Key Laboratory of Neonatal Disease, Ministry of Health, 201102, Shanghai, China.; 3 Department of Anesthesiology, Shanghai Jiao Tong University Affiliated Sixth People's Hospital, Shanghai, 200233, China.; 4 Key Laboratory of Metabolism and Molecular Medicine, Ministry of Education, Department of Biochemistry and Molecular Biology, Institute of Biomedical Sciences, Collaborative Innovation Center of Genetics and Development, School of Basic Medical Sciences, Fudan University, Shanghai, 200032, China.; 5 Department of Medical Imaging, Nanjing Hospital of Chinese Medicine Affiliated to Nanjing University of Chinese Medicine, Nanjing, 210001, China.; 6 ENT institute, Department of Facial Plastic and Reconstructive Surgery, Eye & ENT Hospital, Fudan University, Shanghai, 200031, China.

**Keywords:** neuroblastoma, lncRNA, autophagy, epithelial-mesenchymal transition, ubiquitination

## Abstract

Our previous studies demonstrated that MEG3 was significantly downregulated in neuroblastoma (NB) and its expression was negatively associated with the INSS stage. Overexpression of MEG3 promoted apoptosis and inhibited proliferation in NB cells. In this study, we discovered more potential functions and molecular mechanisms of MEG3 in NB. According to the database, MEG3 positively correlated with the NB survival rate and was negatively associated with malignant clinical features. Moreover, we determined that MEG3 was mainly located in the nucleus by nuclear-cytoplasmic separation and RNA fish assays. Upregulation of MEG3 in stably transfected cell lines was accomplished, and CCK8, colony formation, and EDU assays were performed, which indicated that MEG3 significantly suppressed cell proliferation. Both wound healing and transwell experiments demonstrated that MEG3 decreased cell migration and invasion. CHIRP enrichments showed the anticancer effects of MEG3 were probably linked to autophagy and the mTOR signaling pathway. LC3 fluorescence dots and western blots showed that MEG3 attenuated autophagy by inhibiting FOXO1, but not the mTOR signaling pathway. Furthermore, MEG3 inhibited metastasis through epithelial-mesenchymal transition via the mTOR signaling pathway. Consistent with the above results, downregulation of MEG3 facilitated NB malignant phenotypes. Mechanistically, MEG3 and EZH2 regulated each other via a negative feedback loop and promoted NB progression together. In conclusion, our findings suggested that MEG3 was a tumor suppressor in NB and could be a potential target for NB treatment in the future.

## Introduction

Neuroblastoma (NB) is a malignant tumor originating from sympathetic nerves 1. Tumors are composed of undifferentiated neuroblasts. NB mainly occurs in children with nearly half of NB cases occurring in infants younger than 2 years of age 2, 3. NB has complex clinical manifestations and can occur in different parts of the body 4. The current multidisciplinary joint treatment has significantly improved the cure rate 5. However, NB is generally malignant, and some high-risk NB is still difficult to cure.

Long noncoding RNAs (lncRNAs) are a type of RNAs with transcripts longer than 200 nt 6-8. Generally, lncRNAs do not encode proteins, but regulate gene expression in the form of RNA on the transcriptional, post-transcriptional, and epigenetic levels 9, 10. LncRNAs were originally thought to be “noise” in genome transcription and a byproduct of transcription, which had no specific biological function 11. However, recent studies showed that lncRNAs are involved in important biological processes such as chromatin modification, transcriptional activation, the cell cycle, cell differentiation regulation, and nuclear transport 12-14. Although research on lncRNAs has made rapid progress, the function of most lncRNAs is still unclear.

The maternally expressed MEG3 is the first lncRNA to be found to have tumor suppressor functions 15. Numerous studies show that MEG3 is expressed in a variety of normal tissues, but not in most tumor tissues or tumor cell lines 16-18. MEG3 can stimulate transcriptional activity mediated by the tumor suppressor p53, make p53 protein aggregate in cells, reduce the expression of the p53 negative regulator MDM2 protein, and selectively activate p53 downstream target genes 19, 20. Zhu et al. demonstrated that MEG3 participated in gastric cancer progression and metastasis by regulating the epithelial-mesenchymal transition (EMT) 21. MEG3 promoted glioma cell autophagy and inhibited cell proliferation and migration through positively regulating Sirt7 by inhibition of the PI3K/AKT/mTOR signaling pathway 22. The expression of MEG3 is controlled by epigenetics, and its gene is abnormally CpG-methylated in various tumors 23, 24. Furthermore, Mondal et al. demonstrated that MEG3 and EZH2 shared common target genes 25. Jin et al. showed that MEG3 was associated with EZH2 and decreased its protein level by facilitating its ubiquitination, and MEG3 exerted its functions through EZH2 to regulate downstream target genes 26.

Our previous study indicated that the expression of MEG3 negatively correlated with the NB INSS stage, and that overexpression of MEG3 inhibited proliferation and promoted apoptosis of SK-N-BE(2)-C cells 27. In the present study, we first used a public database to investigate the clinical significance of MEG3. The results indicated that MEG3 was negatively associated with event-free survival and overall survival of NB patients. Low expression of MEG3 in patients had a higher NB risk, advanced International Neuroblastoma Staging System (INSS) stages, and faster disease progression. Furthermore, MEG3 was mainly located in the nucleus. Functional studies showed that MEG3 suppressed cell proliferation, migration, and invasion. Mechanistically, the anticancer effects of MEG3 were probably associated with autophagy via FOXO1, and not the mTOR pathway. Moreover, MEG3 inhibited tumor metastasis through the mTOR-mediated EMT. Furthermore, MEG3 and EZH2 regulated each other by forming a negative feedback loop and together promoted NB progression. Taken together, MEG3 and EZH2 could in the future be joint targets for the treatment of childhood neuroblastomas.

## Materials and Methods

### Human NB cell lines

SH-SY5Y cells were purchased from the Cell Bank of the Chinese Academy of Science (Shanghai, China). SK-N-BE (2)C and SK-N-AS cells were gifts from Professor Kai Li of Fudan University. All cells were cultured in DMEM and F12 (1:1) with 10% fetal bovine serum (FBS) in a humidified incubator with 5% CO_2_ at 37 °C. Cell culture dishes and plates were purchased from Xinyou Biotechnology (Hangzhou, China).

### Nuclear and cytoplasmic extract preparation

The cell density reached 80%-90% confluency in 10-cm dishes, and cells were washed three times using precooled PBS. Then, 0.5 mL cytoplasmic extraction buffer (Invent Biotechnologies, Inc) was added to the dishes on ice for 10 min. Lysates were transferred to tubes and shaken vigorously for 30 s. After centrifuging at 12,000 × *rpm* for 15 min at 4 °C, TRIzol was added to the pellet and supernatant for RNA extraction. For RNA fish assays, MEG3 probe and relative control probes 18S and U6 were generated by RiboBio (Guangzhou, China) and detailed protocols were performed as previously described 28.

### Construction of stably transfected cell lines

MEG3 overexpression plasmids were constructed in the pcDH vector by Genomeditech (Shanghai, China). Lentivirus packaging was in 293T cells with l-pei. Following viral infection and puromycin screening, stably transfected cells were acquired. The Smart Silencer (mix of siRNA and ASO) of MEG3 was purchased from RiboBio (Guangzhou, China). Smart Silencer was transiently transfected with Lipofectamine 3000 (Invitrogen, USA) for 48 h and then was used for subsequent assays.

### Quantitative real time-polymerase chain reaction (qRT-PCR)

Total cell RNA was extracted with TRIzol reagent (Takara, Japan) and the cDNA was synthesized using cDNA Reverse Transcription kits (Takara). The protocol was carried out at 42℃ for 2 min to degrade gDNA, followed by 37℃ for 15 min and 85 °C for 5 s for reverse transcription. QRT-PCR was performed using SYBR Green PCR Master Mix (Takara) with a Roche machine. The PCR parameters were: initial denaturation at 95 °C for 5 min, followed by 35 cycles at 95 °C for 30 s, 58 °C for 30 s, and 72 °C for 30 s. GAPDH was the internal control. Primers were: MEG3-forward, 5ʹ-CCTCACCTCCAATTTCCTCTTC-3ʹ, MEG3-reverse, 5ʹ-TCCAGCAGCTAACCTCATTAAC-3ʹ; GAPDH-forward 5'-GGAGCGAGATCCCTCCAAAAT-3', GAPDH-reverse, 5ʹ-GGCTGTTGTCATACTTCTCATGG-3'. Relative gene expression was analyzed by the 2-ΔΔCt method and data were analyzed using GraphPad Prism 5 software.

### Cell proliferation assays

The proliferation of NB cells was detected using CCK-8 kits (Yeasen, Shanghai, China) in accordance with the manufacturer's instructions. Cells were cultured in 96-well cell culture plates with 1,000 cells in 100 µL medium, and were detected by a microplate reader with 10 µL CCK8 reagent for 2.5 h. For EDU assays, cells were treated with 50 µM EDU for 2 h, followed by staining according to instructions (RiboBio, Guangzhou, China). For cloning formation experiments, 1,000 cells per well were cultured in 6-well cell culture plates for 14 days. After fixing using 4% paraformaldehyde, the cells were stained with 0.25% Crystal Violet for 45 min.

### Wound healing assays

Cells were cultured in 6-well cell culture plates. After the cells were attached, a gap was made using a tip. The gap was observed at 100× magnification and photographs were taken using a microscope (Olympus, Japan) at 0 h, 24 h, and 48 h.

### Cell migration and invasion assays

The 24-well cell culture plates with 8-μm micropore inserts were used for cell migration and invasion assays. For cell migration assays, 1 × 10^5^ cells were seeded into upper wells without FBS. In the cell invasion experiments, upper wells were coated with 50 μL Matrigel (Becton, Dickinson) diluted five times before 2 × 10^5^ cells were seeded into upper wells without FBS. After 48 h, the cells were fixed with 4% paraformaldehyde and stained with 0.25% Crystal Violet for 45 min.

### Western blots

Cell proteins were extracted with protein lysis buffer (Beyotime Institute of Biotechnology) and boiled with 1× loading buffer at 105 °C for 10 min. After electrophoresis and transfer, the membrane was blocked with 8% skimmed milk. After 1 h, the membrane was incubated with primary antibodies overnight at 4 °C. After washing four times with TBST, membranes were incubated with secondary antibodies for 1 h at room temperature. Signals of proteins were detected with Enhanced Chemiluminescent Reagent kits (New Cell & Molecular Biotech, Suzhou, China).

### Chromatin isolation by RNA purification (CHIRP)

MEG3 sense and antisense DNA probes, and β‑galactosidase sense and antisense DNA probes were all designed by an online probe designer (singlemoleculefish.com). Oligonucleotides were biotinylated at the 3' end with an 18‑carbon spacer arm. The cells were collected and subjected to CHIRP, as described by Chu et al. (29). GO and KEGG analyses were performed using the DAVID Functional Annotation web-based tool (http://david.ncifcrf.gov).

### Statistical analysis

All assays were repeated independently at least three times. All results are presented as the mean ± standard deviation (SD). Groups were compared using Student's *t*-test or one-way ANOVA. *P* < 0.05 was considered statistically significant.

## Results

### MEG3 was closely related to clinical features

**Figure 1:**
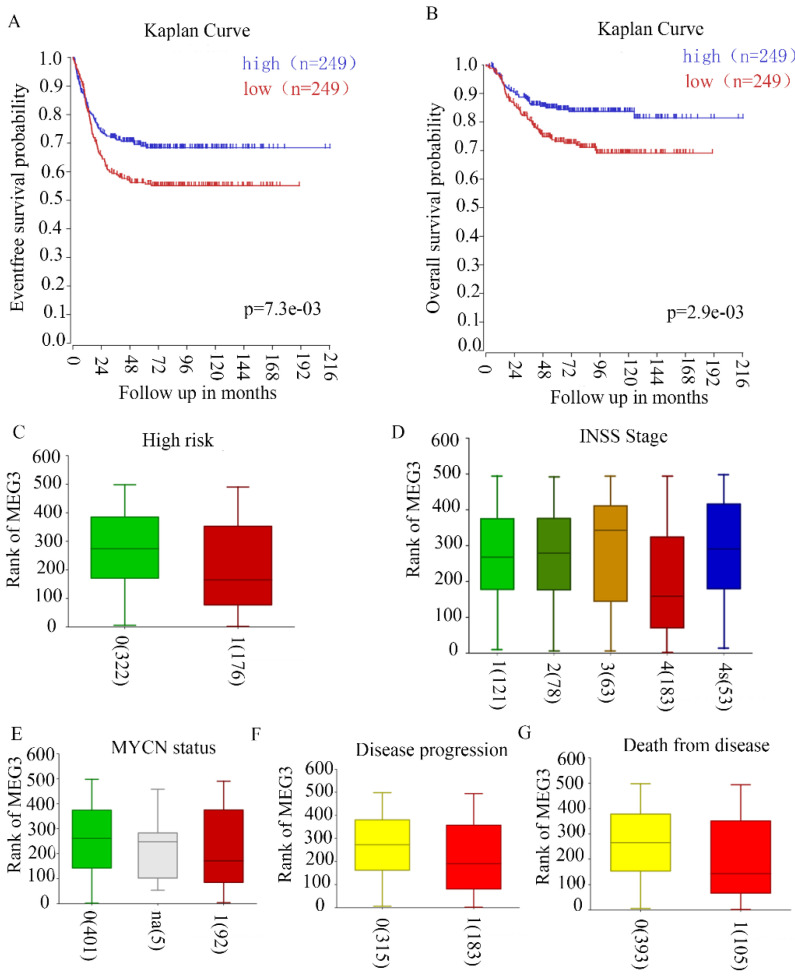
** MEG3 was associated with neuroblastoma (NB) with malignant clinical features.** A, B. MEG3 expression was related to the 5-year event-free survival rate and overall survival rate. Patients with higher MEG3 levels had longer lifetimes. C, Relationship of high risk NB and MEG3 expression: 0, not high risk; 1 high risk, 0 vs. 1, p = 1.7e^-3^. D, Relationship of NB INSS stage and MEG3 expression: 1, 2, 3, 4, 4s represent different NB stages, 1 vs. 4, p = 5.0e^-3^, 2 vs. 4, p = 4.8e^-3^, 3 vs. 4 p = 1.8e^-3^, 4 vs. 4s, p = 4.3e^-3^. E, Relationship of MYCN status and MEG3 expression: 0, MYCN no amplification; 1, MYCN amplification, 0 vs. 1, p = 0.48. F, Relationship of NB progression and MEG3 expression: 0, no disease progression, 1, disease progression, 0 vs 1, p = 1.8e^-3^. G, Relationship of cause of death and MEG3 expression; 0, no death from NB; 1, death from NB, 0 vs 1, *p* = 6.8e^-4^.

Our previous study revealed that MEG3 negatively correlated with INSS NB stages in 32 tissues of patients in our hospital. To further confirm the relationship between MEG3 and clinical characteristics, R2 genomic analysis and a visualization platform (http://r2.amc.nl) were used. The website is based on genomic analyses and visualization applications. The R2 database contains mRNA gene expression profiles for more than 70,000 individual human samples. The samples are grouped in so-called datasets, and each dataset has its own characteristics such as tissue type, tumor type, or data from cell line experiments. A GEO dataset (GSE62564) analysis was performed for these data. Patients with high MEG3 expression had higher 5-year event-free survival probabilities and overall survival probabilities (Figure 1A & B). High risk NB patients had lower expression of MEG3 (Figure 1C). MEG3 expression was closely related to disease stages. Furthermore, patients with stage IV NB had a lower expression of MEG3 compared with patients with stage I, II, and III. However, patients with stage IV disease had higher expression of MEG3 than patients with stage III disease (Figure 1D). In addition, lower expression of MEG3 also was directly correlated with disease progression and cause of death, but not the MYCN amplification status (Figure 1E-G).

### MEG3 was mainly located at cell nuclei and inhibited cell proliferation

**Figure 2:**
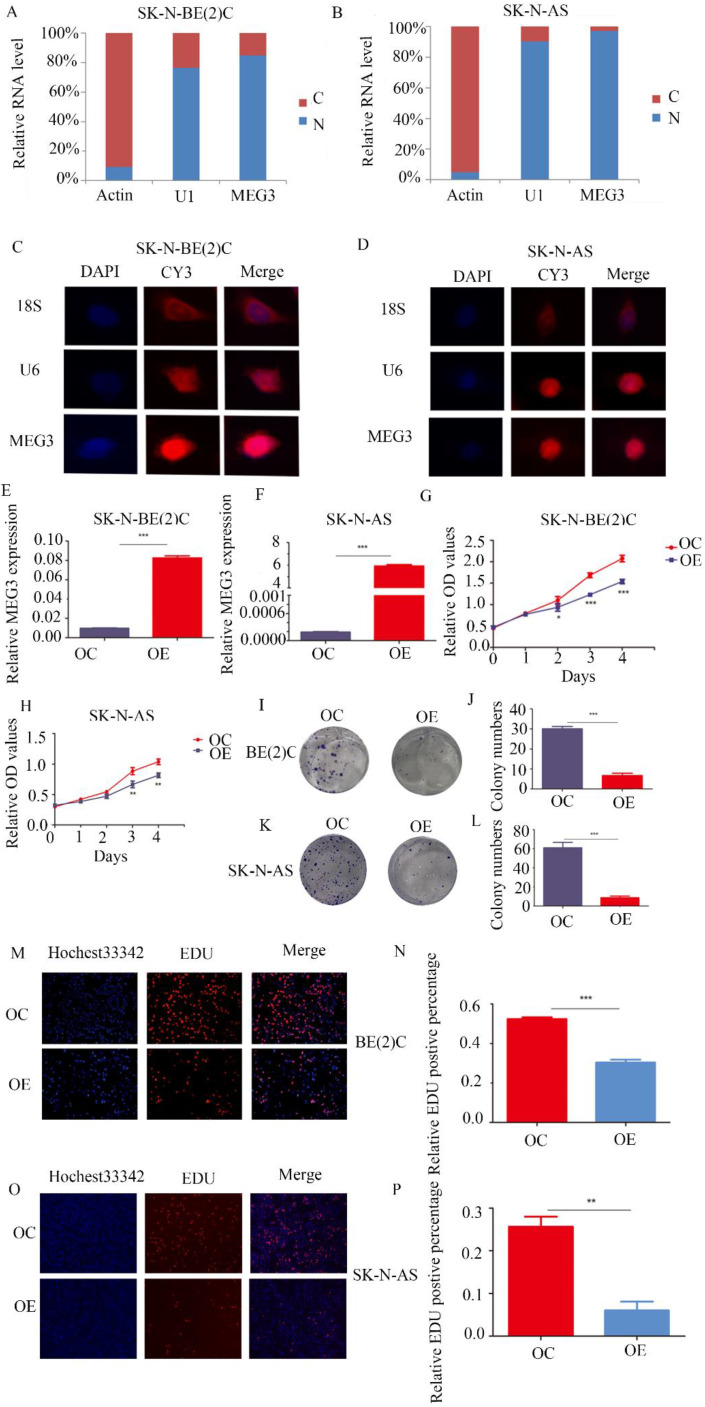
** MEG3 inhibited neuroblastoma (NB) cell proliferation.** A, B. Nuclear-cytoplasmic separation assays for NB cells SK-N-BE (2)C and SK-N-AS indicated that MEG3 was mainly located in the nucleus, similar to U1 (actin was used for the cytoplasmic internal control, and U1 was used for the nuclear internal control). C, D. RNA fluorescence *in situ* hybridization for SK-N-BE (2) C and SK-N-AS cells also showed that MEG3 was mainly located in the nucleus. DAPI (blue) represented nuclei, CY3 (red) represented different kinds of probes (18S was a cytoplasmic internal control, and U6 was a nuclear internal control). E, F. QPCR was performed to detect the efficiency of MEG3 overexpression in SK-N-BE (2)C and SK-N-AS cells. G-P. The effects of MEG3 on cell proliferation were tested by the CCK-8 (G, H), and colony formation assay (I, J, K, L), and by EDU incorporation (M, N, O, P). ^**^P < 0.01; ^***^P < 0.005.

MEG3 was differentially expressed in various kinds of NB cell lines (Figure S1A). To further determine the function of MEG3, understanding its expression and location was necessary. Both nuclear-cytoplasmic separation (Figure 2A, B) and RNA fluorescence *in situ* hybridization (Figure 2C, D; Figure S1B) indicated that MEG3 was mainly located in cell nuclei. Consequently, MEG3 was overexpressed in stably transfected NB cell lines SK-N-BE (2)C, SK-N-AS, and SH-SY5Y (Figure 2E, F; Figure S1C). To analyze proliferation, we used CCK-8 and colony formation assays, and found that upregulation of MEG3 inhibited cell proliferation (Figure 2G, H; Figure S1F) and decreased cell colony formation (Figure 2I-L). To further verify the negative influence of MEG3 on cell proliferation, the EDU assay was performed in three NB cell lines (Figure 2M-P; Figure S1D, E). The results also showed that cell proliferation was inhibited with MEG3 overexpression. Taken together, these results demonstrated that MEG3 significantly inhibited NB cell proliferation.

### MEG3 inhibited cell migration and invasion *in vitro*

**Figure 3:**
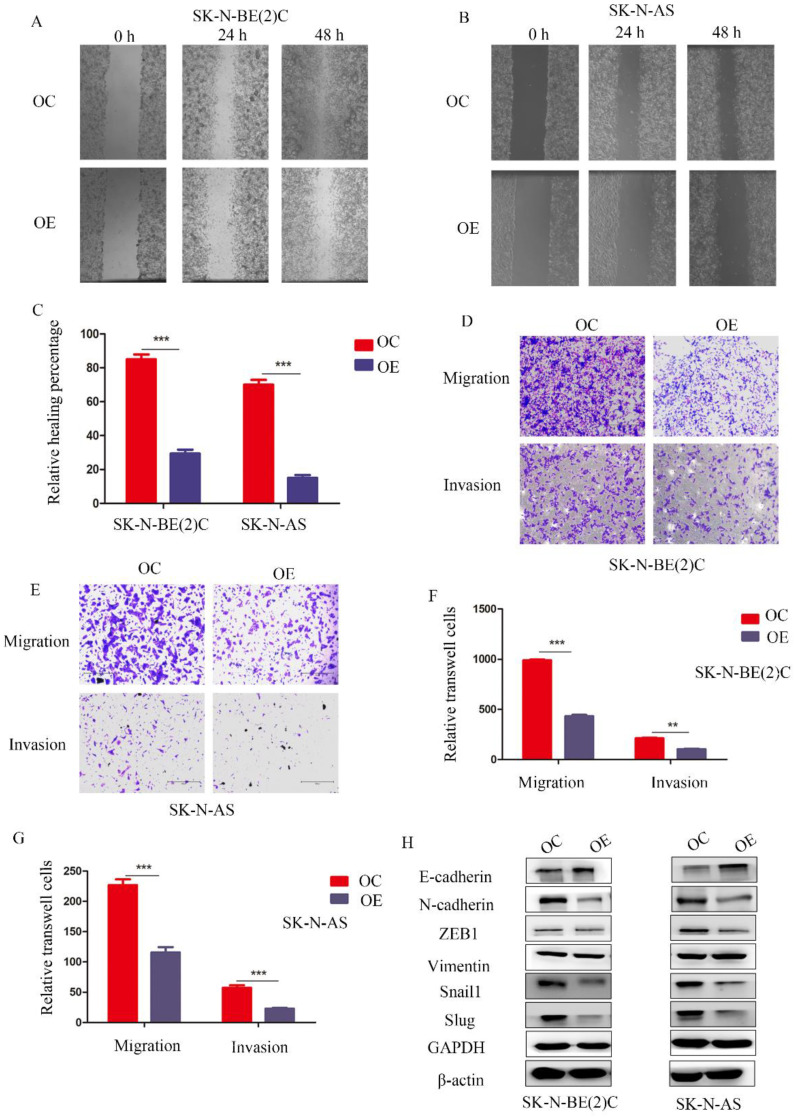
** MEG3 suppressed NB cell migration and invasion by affecting EMT.** A, B. Wound healing assays for MEG3 overexpression and control groups in SK-N-BE (2)C and SK-N-AS cells indicated that MEG3 inhibited cell migration. C. Statistics of the percentage of wound healing after 48 h, D, E. Overexpression of MEG3 significantly decreased cell migration and invasion in both SK-N-BE (2)C and SK-N-AS cells when compared with the control groups. F, G. Statistics of migration and invasion cells in the Transwell assay after treatment for 48 h. H. Western blots indicated that MEG3 overexpression significantly inhibited the EMT marker proteins in SK-N-BE (2)C and SK-N-AS cells.

To study the function of MEG3 in migration and invasion of NB cells, wound healing assays and Transwell experiments were performed, respectively. The wound healing assay showed that overexpression of MEG3 inhibited cell migration (Figure 3A-C). Similarly, Transwell experiments indicated that MEG3 repressed cell migration and invasion (Figure 3D-G; Figure S1G). Furthermore, MEG3 might inhibit migration and invasion by participating in the EMT (Figure 3H, Figure S2A). Overall, our data showed that MEG3 inhibited EMT-mediated migration and invasion in NB cells.

### CHIRP for MEG3

**Figure 4:**
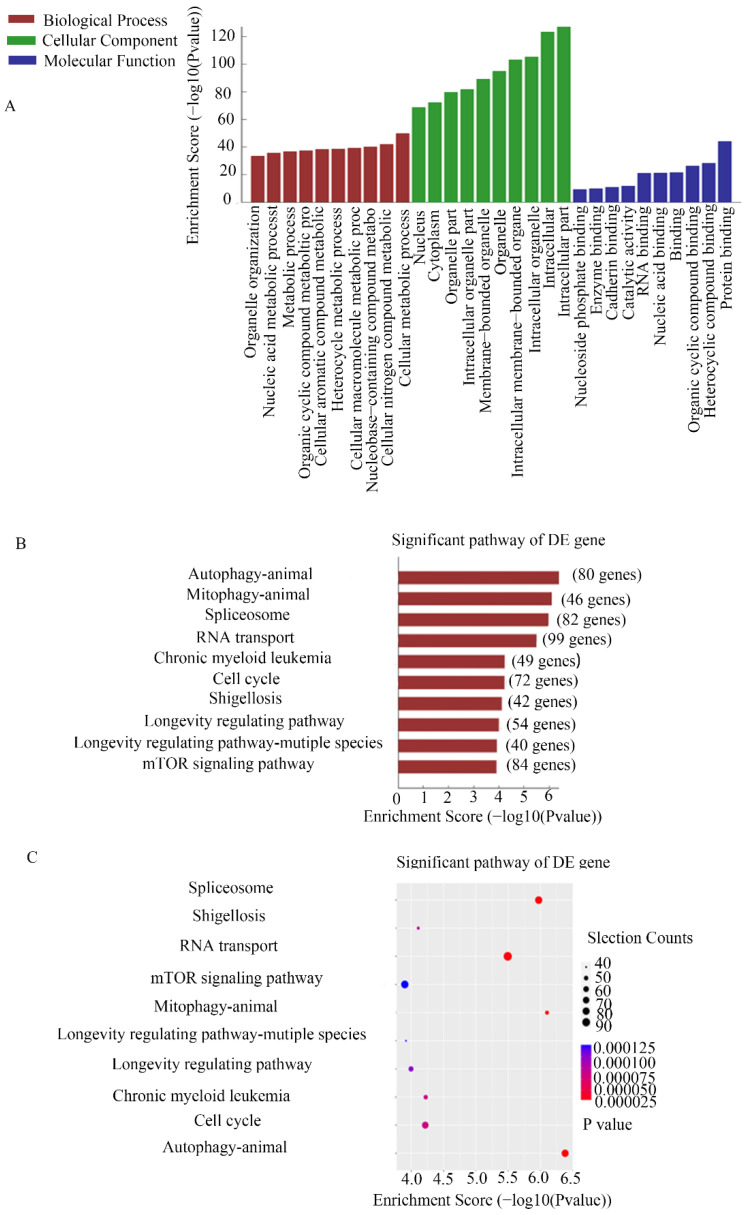
** CHIRP analysis for enriched DNA binding with MEG3 in NB cells.** A. GO analysis for CHIRP results showed that MEG3 may participate in various biological processes. B, C. KEGG analysis for CHIRP results indicated that MEG3 was associated with autophagy, the mTOR signaling pathway, and the cell cycle.

CHIRP assays were used to study the molecular mechanism of MEG3 in NB. Thousands of genes were shown to interact with MEG3. For further analysis, we selected genes enriched in the promoter region using a MEG3 probe. GO analysis showed that MEG3 may be involved in biological processes such as cell metabolism process and catalytic activity (Figure 4A). KEGG pathway analysis demonstrated that MEG3 was connected to animal autophagy and the mTOR signaling pathway (Figure 4B-C). It is well-known that mTOR is a classic autophagy regulatory pathway. Whether MEG3 modulated autophagy via a typical mTOR pathway in NB was further studied.

### MEG3 did not inhibit autophagy through the mTOR pathway

**Figure 5:**
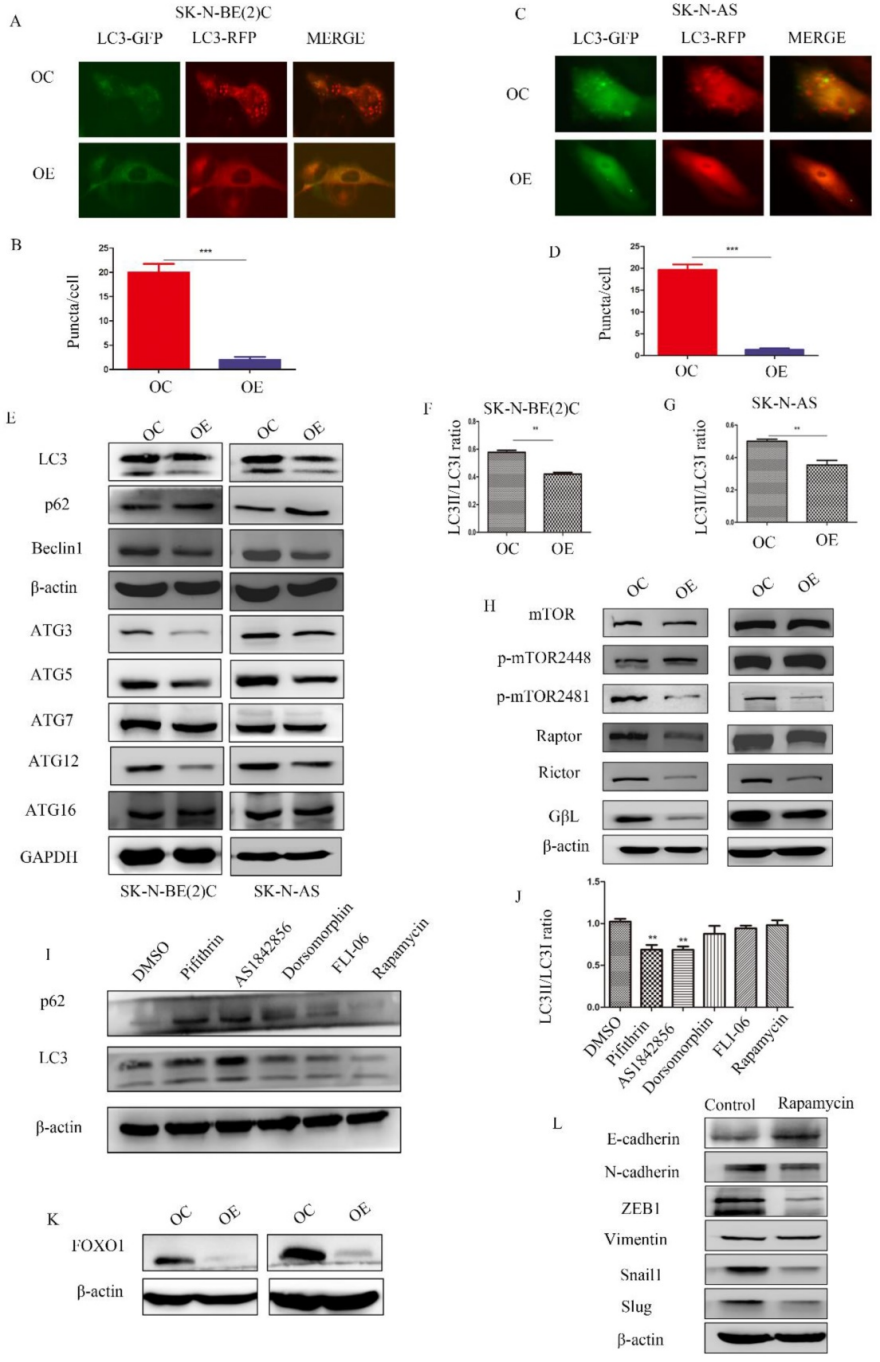
** MEG3 attenuated NB cell autophagy via inhibition of the FOXO1 signaling pathway.** A-D. MEG3 overexpression attenuated cell autophagy in SK-N-BE (2)C cells (A, B), and SK-N-AS cells (C, D)., GFP^+^RFP^+^ yellow puncta represented autophagosomes. E. Western blots for autophagy markers showed that upregulation of MEG3 decreased the LC3 II/LC3 I ratio, Beclin1, ATG3, ATG5, and ATG12 levels, but increased autophagy substrate p62 levels. F, G. Grayscale of the LC3II/LC3I ratio using ImageJ software in SK-N-BE (2)C and SK-N-AS. H. Western blots for the mTOR signaling pathway proteins demonstrated that MEG3 overexpression inhibited the mTOR signaling pathway. I. The LC3 II/LC3 I ratio was decreased while p62 expression was increased after treatment with inhibitors of p53 (25 µm Pifithrin) and FOXO1 (30 nm AS1842856), but not AMPK (100 nm Dorsomorphin), Notch (2.5 µm FLI-06), or mTOR (5 µm Rapamycin,) after 24 h. J. Grayscale of the LC3II/LC3I ratio using ImageJ software in SK-N-BE (2)C treated with different inhibitors. K. Western blots indicated that FOXO1 was inhibited in the MEG3 overexpression groups when compared to the control groups. L. The EMT marker proteins were significantly decreased after treatment of SK-N-BE (2)C cells with 5 µm rapamycin for 24 h. ^**^P < 0.01; ^***^P < 0.005.

To determine the relationship among MEG3, mTOR, and autophagy, RFP-GFP-LC3 virus (Genomeditech) was used to construct stably transfected cells to observe autophagosomes in NB cells. Overexpression of MEG3 suppressed autophagy at an early stage (Figure 5A-D). Protein levels of autophagy markers also indicated that upregulation of MEG3 inhibited autophagy (Figure 5E-G, Figure S2 B). In our results, the mTOR signaling pathway was inactivated by MEG3 upregulation (Figure 5H, Figure S2C). It showed that MEG3 did not affect autophagy through the mTOR signaling pathway because it is well-known that inhibition of mTOR increases autophagy. To further characterize the putative mechanism involved in autophagy, we used inhibitors of all pathways enriched in CHIRP. Therefore, inhibitors of p53 (pifithrin), FOXO1 (AS1842856), AMPK (dorsomorphin), and Notch (FLI-06) pathways were used to detect relative autophagy proteins, lc3 and p62. We found that autophagy significantly decreased with the FOXO1 inhibitor, AS1842856 (Figure 5I-J). Furthermore, upregulation of MEG3 also suppressed FOXO1 expression (Figure 5K). Rapamycin, an mTOR pathway inhibitor, did not affect autophagy in NB, but subsequent results showed it significantly inhibited EMT (Figure 5L).

### Silencing MEG3 promoted NB malignant behavior

**Figure 6:**
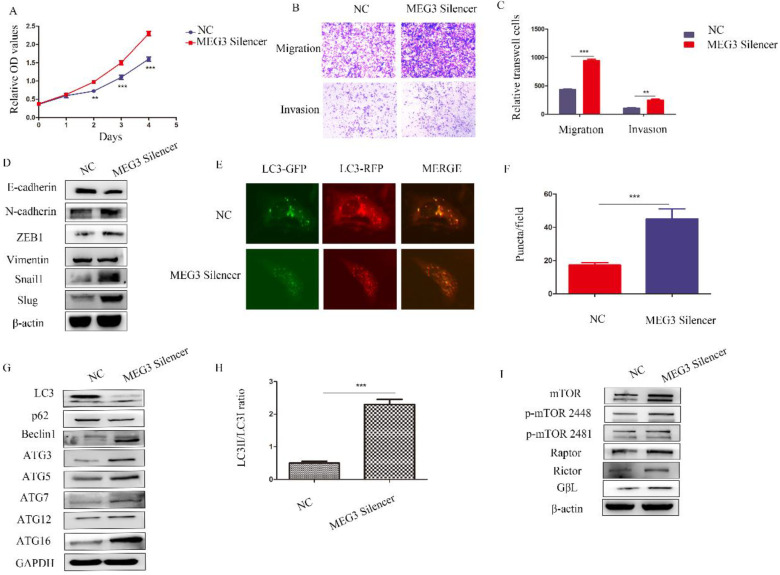
** Downregulation of MEG3 inhibited SK-N-BE (2)C cell proliferation, metastasis, and autophagy.** A. CCK8 assays showed downregulation of MEG3 promoted cell proliferation after treatment with the MEG3 silencer and negative control for 48 h. B, C. Silencing of MEG3 increased cell migration and invasion as shown using the Transwell assay. D. Knockdown of MEG3 promoted the EMT. E, F. MEG3 downregulation increased cell autophagy in SK-N-BE (2)C cells. GFP+RFP+ yellow puncta represented autophagosomes. G. Western blots showed that silencing of MEG3 increased autophagy marker expression after MEG3 silencing and control treatment for 48 h. F. RFP-GFP-LC3 for MEG3 silencing and control groups of SK-N-BE (2)C cells (GFP^+^RFP^+^ yellow puncta represented autophagosomes). H. Grayscale of the LC3II/LC3I ratio using ImageJ software in the MEG3 silencing and control groups of SK-N-BE (2)C cells. I. Western blots showed that the mTOR signaling pathway was inhibited by MEG3 silencing for 48 h. ^**^P < 0.01; ^***^P < 0.005.

To further study the biological roles of MEG3, silencing of MEG3 was performed in SK-N-BE (2)C cells, which had the highest level of MEG3. Consistent with the above results, downregulation of MEG3 promoted cell proliferation (Figure 6A). Furthermore, silencing MEG3 increased cell migration and invasion (Figure 6B-C) by promoting the EMT (Figure 6D). RFP-GFP-LC3 fluorescence demonstrated that silencing of MEG3 increased autophagy (Figure 6E-F), while downregulation of MEG3 increased autophagy marker levels (Figure 6G-H) and activated the mTOR signaling pathway (Figure 6I).

### MEG3 and EZH2 were mutually regulated via a negative feedback loop

**Figure 7:**
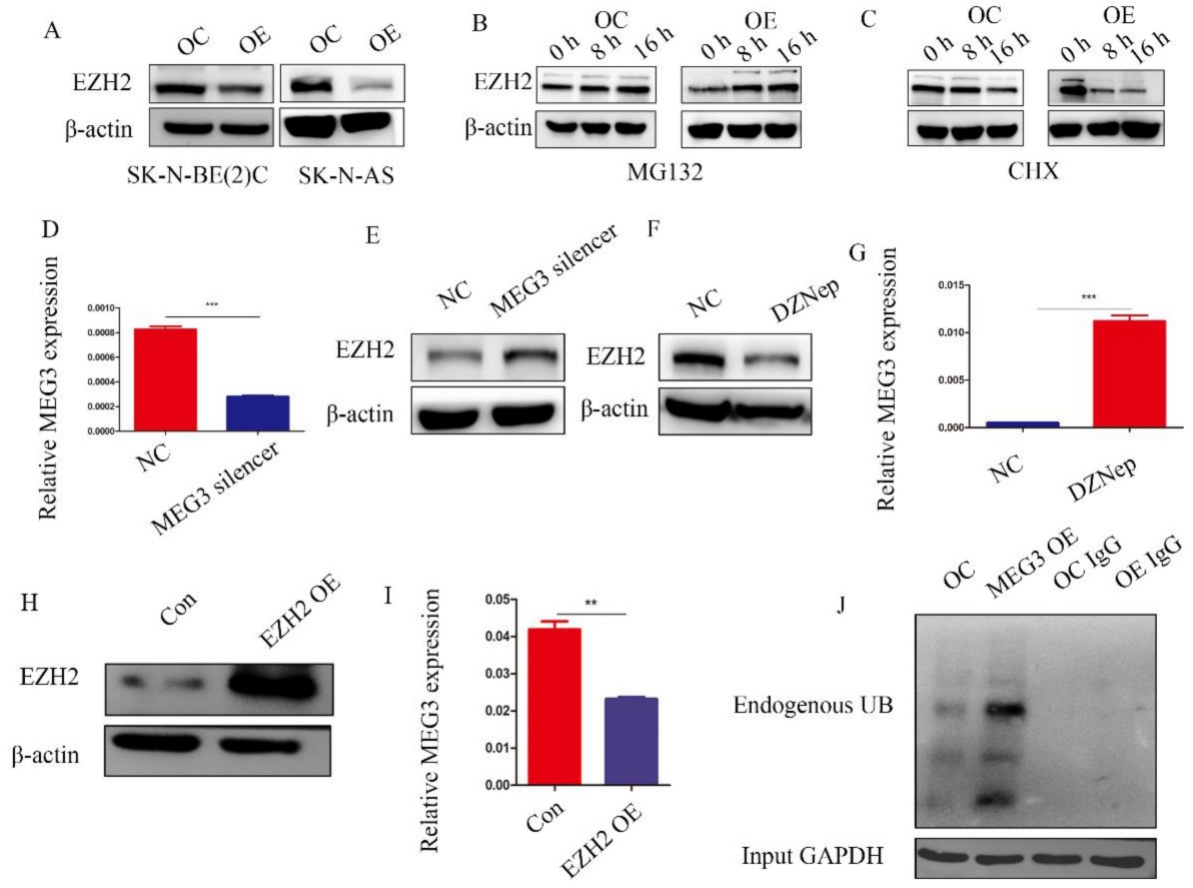
** MEG3 and EZH2 regulated each other via a negative feedback loop.** A, Overexpression of MEG3 significantly decreased EZH2 expression in SK-N-BE (2)C and SK-N-AS cells. B, EZH2 expression was increased in the MEG3 overexpression group after treatment of SK-N-BE (2)C cells with 50 µm MG132 at 0, 8, and 16 h. C. EZH2 was decreased more in the MEG3 overexpression group than the control group after treatment of SK-N-BE (2)C cells with 0.1 µm cycloheximide (CHX) at 0, 8, and 16 h. D. The qRT-PCR verified the MEG3 silencer knockdown efficiency in SK-N-BE (2)C cells treated for 48 h. E. Downregulation of MEG3 promoted EZH2 levels in SK-N-BE(2)C cells treated with silencing for 48 h. F. Ten µm DZNep for 48 h significantly inhibited EZH2 protein levels. G. QPCR showed that MEG3 expression was increased after treatment of SK-N-BE(2)C cells with 10 µm DZNep for 48 h. H. Western blotting showed that EZH2 was successfully overexpressed in SK-N-BE (2)C cells. I. Overexpression of EZH2 decreased MEG3 expression in SK-N-BE (2)C cells. J. Overexpression of MEG3 promoted ubiquitin binding with EZH2 in SK-N-BE (2)C cells. ^***^P < 0.005.

Previous studies have reported that MEG3 was associated with EZH2, and that EZH2 was a tumor target, which acted as an oncogene in NB. Characterizing the regulation between MEG3 and EZH2 in NB showed that overexpression of MEG3 decreased EZH2 expression (Figure 7A). Inhibiting ubiquitination degradation with MG132 treatment in the MEG3 overexpression group significantly increased EZH2 levels when compared to the control group (Figure 7B). Moreover, overexpression of MEG3 caused reduced EZH2 stability (Figure 7C). Conversely, silencing MEG3 increased EZH2 expression (Figure 7D-E). The EZH2 inhibitor, DZNep, increased MEG3 levels (Figure 7F-G) and overexpression of EZH2 inhibited MEG3 expression (Figure 7H-I). In addition, upregulation of MEG3 increased EZH2 ubiquitination (Figure 7J). Collectively, the results showed that MEG3 and EZH2 may form a negative feedback loop and regulate each other (Graphical Abstract).

## Discussion

Previous studies found numerous lncRNAs through microarray and RNA-seq that are crucial for the development of various diseases, especially malignant tumors 30-32. According to the NCBI database, MEG3 is located on chromosome 14q32.3 in *Homo sapiens* and functions as a tumor suppressor in many cancers 33, 34. Although MEG3 was reported to be involved in tumorigenesis and development of various cancers, the function and molecular mechanism of MEG3 in NB remains unclear.

In the present study, we investigated MEG3 downregulation in NB patients. Overexpression of MEG3 in NB cells suppressed cell proliferation, migration, and invasion. Furthermore, upregulation of MEG3 inhibited autophagy. It is known that mTOR exists in two complexes, mTORC1 and mTORC2. They are different in subunit composition and function 35. The mTORC1 is a central regulator of cell growth and nutrient sensing, and activation of mTORC1 inhibits autophagy, while it promotes cell growth 36. The mTORC1 was discovered earlier than mTORC2, and its activity can be inhibited by rapamycin, but the activity of the mTORC2 could not be inhibited by rapamycin, and was mainly involved in the synthesis of cytoskeletal proteins 35. In our study, both Raptor (mTORC1) and Rictor (mTORC2) were inhibited by MEG3 overexpression. Phospho-mTOR (Ser2481) and GβL (common components of both complexes were also suppressed. We therefore assumed that MEG3 inhibited both mTORC1 and mTORC2. In the present results, overexpression of MEG3 suppressed mTORC1 and cell growth, but did not promote autophagy. This was contrary to the classical view that inhibition of the mTOR pathway promotes autophagy. It suggested that MEG3 affected autophagy, independent of mTOR. To characterize the pathway involved in MEG3 and autophagy, we used five autophagy relative pathway inhibitors. These five pathways were also enriched by MEG3 CHIRP. The results showed that the mTOR signaling pathway inhibitor, rapamycin, had no significant effect on NB cell autophagy. However, the FOXO1 inhibitor suppressed autophagy. We therefore concluded that MEG3 decreased autophagy through FOXO1, but not mTOR. Furthermore, our study indicated that MEG3 inhibited NB metastasis by inhibiting the EMT via suppression of the mTOR signaling pathway. In previous studies, MEG3 promoted or inhibited autophagy in various cancers. In human glioma cells, upregulation of MEG3 inhibited cell proliferation while increasing cell apoptosis and autophagy. MEG3 decreased cell proliferation and migration but promoted autophagy in U251 cells by positively regulating Sirt7 by inhibiting the PI3K/AKT/mTOR signaling pathway 22. However, MEG3 was involved in autophagy inhibition, which is involved in adenosine-induced cytotoxicity in HepG2 cells 38. The cytotoxicity was synergistic with overexpression of MEG3, via downregulation of ILF3, which regulated activation of the PI3K/AKT/mTOR pathway and inactivation of the beclin-1 signaling pathway 38. Furthermore, MEG3 is expressed in low amounts in chemotherapy-sensitive lung cancer tissues, and upregulation of MEG3 inhibits autophagy by increasing the sensitivity to vincristine chemotherapy 39. The relationship between autophagy and tumors is complicated. Normal cell autophagy enhancement can suppress tumorigenesis, but tumor cells can increase autophagy to inhibit a series of stress reactions involving hypoxia and chemotherapy.

The polycomb repressor complex 2 (PRC2) core component molecule EZH2 has been reported to be important in biological processes, such as the cell cycle, cell differentiation, and carcinoma progression 40, 41. Previous studies showed that EZH2 was significantly increased in NB and negatively associated with NB prognosis 42. EZH2 regulated NB cell proliferation, apoptosis, metastasis and differentiation 43-45. It had been shown that various lncRNAs interact with EZH2 46. MEG3 is a lncRNA closely related to EZH2, and MEG3 attenuates EZH2 by increasing its ubiquitination in gallbladder cancer 26. Furthermore, MEG3 facilitates H3K27 trimethylation of EN2 through binding with EZH2, thus suppressing the development of prostate cancer 47. Our findings also showed that MEG3 reduced EZH2 expression by increasing ubiquitination degradation. In addition, EZH2 inhibited the expression of many tumor suppressor genes through H3K27me3. The present study also found that EZH2 suppressed MEG3 expression, which was possibly modulated via H3K27me3. Therefore, MEG3 and EZH2 may form a negative feedback loop to promote NB development. Moreover, the loop was a malignant cycle, which aggravated the effects of EZH2 and MEG3.

In conclusion, numerous studies have shown that MEG3 is a new type of lncRNA tumor suppressor. Our study also indicated that MEG3 exerted a strong anticancer effect in NB. Although the detailed molecular mechanism of MEG3 is still limited, MEG3 may become a target for clinical treatment of NB in the near future.

## Supplementary Material

Supplementary figures and tables.

## Abbreviations

NB: neuroblastoma
LncRNA: long non-coding RNA
DMEM: Dulbecco's modified eagle's medium
PBS: phosphate-buffered saline
FBS: fetal bovine serum
QRT-PCR: quantitative real-time PCR
GAPDH: glyceraldehyde 3-phosphate dehydrogenase
CCK8: cell counting kit-8
TBST: tris-buffered saline with Tween 20
EMT: epithelial-mesenchymal transition
INSS: international neuroblastoma staging system
EDU: 5-ethynyl-2ʹ-deoxyuridine
