# Supplementary material for: Downregulation of MEG3 promotes neuroblastoma development through FOXO1-mediated autophagy and mTOR-mediated epithelial-mesenchymal transition
Source: Int J Biol Sci. 2020 Oct 3;16(15):3050–61. doi: 10.7150/ijbs.48126 (PMC7545718; doi:10.7150/ijbs.48126)
Supplement: Supplementary file 1 — Supplementary figures and tables. [file ijbsv16p3050s1.pdf]

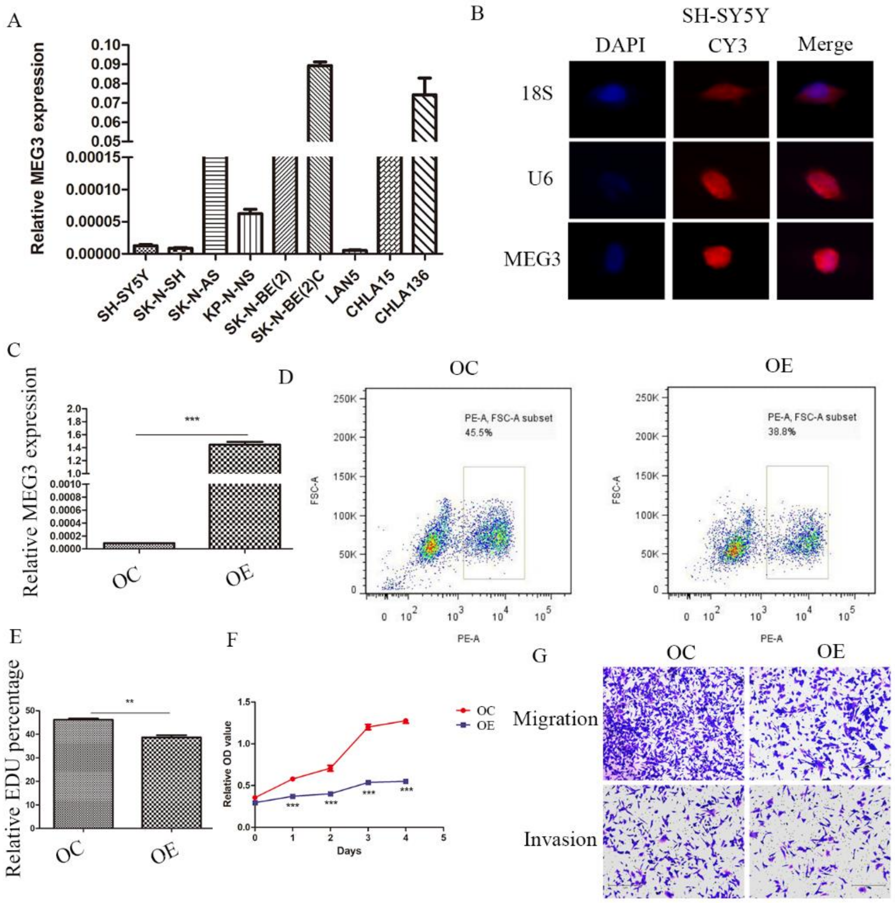


**Figure S1 MEG3 inhibited SH-SY5Y cell proliferation, migration and invasion.**

A. The qRT-PCR for MEG3 expression in different NB cell lines. B. RNA fluorescence in situ hybridization for SH-SY5Y cells. C. The qRT-PCR for MEG3 overexpression in SH-SY5Y cells. D, E. EDU assays for MEG3 overexpression and control groups in SH-SY5Y cells. F. CCK-8 assays for MEG3 overexpression and control groups in SH-SY5Y cells. G. Transwell assays for MEG3 overexpression and control groups in SH-SY5Y cells. **P < 0.01; ***P < 0.005.

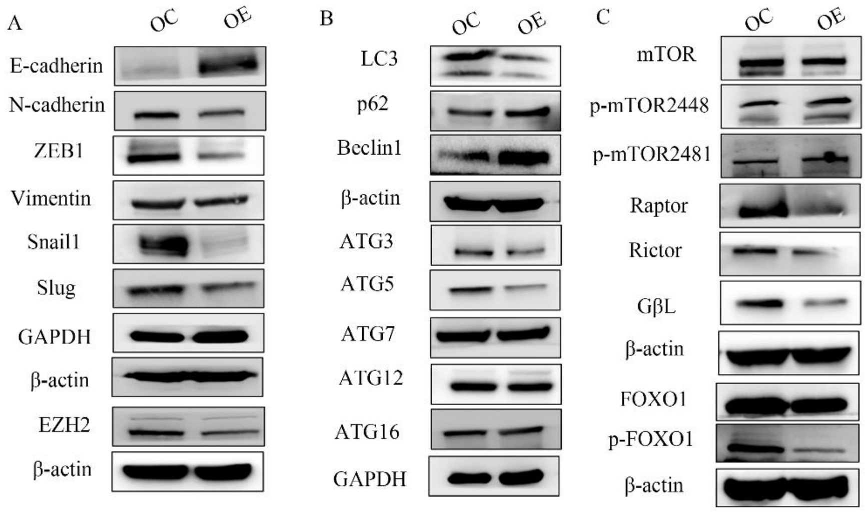


**Figure S2 MEG3 inhibited the EMT, autophagy, and the mTOR signaling pathway in SH-SY5Y cells.** A. Western blots for EMT markers in the MEG3 overexpression and control groups. B. Western blots for autophagy markers in the MEG3 overexpression and control groups. C. Western blots for mTOR signaling pathway proteins in the MEG3 overexpression and control groups.

**TableS1 Antibody information**

| Antibody | Vendor | Catalog number |
|---|---|---|
| E-cadherin | CST | 3195T |
| N-cadherin | Proteintech | 22018-1-AP |
| ZEB1 | Proteintech | 21544-1-AP |
| Vimentin | Santa Cruz | Sc-6206 |
| Snail1 | CST | 3879S |
| Slug | Santa Cruz | Sc-166476 |
| GAPDH | Proteintech | 60004-1-Ig |
| β-Actin | Proteintech | 60008-1-Ig |
| LC3 | CST | 12741S |
| P62 | Santa Cruz | Sc-28359 |
| Beclin1 | CST | 3495S |
| ATG3 | CST | 3415S |
| ATG5 | CST | 12994S |
| ATG7 | CST | 8558S |
| ATG12 | CST | 4180S |
| ATG16 | CST | 8089S |
| mTOR | CST | 2983S |
| p-mTOR2448 | CST | 5536S |
| p-mTOR2481 | CST | 2974S |
| Raptor | CST | 2280S |
| Rictor | CST | 2114S |
| GβL | CST | 3274S |
| FOXO1 | CST | 2880S |
| EZH2 | CST | 5246S |
| Ub | Santa Cruz | Sc-166553 |
